# Does Obesity Cause Thyroid Cancer? A Mendelian Randomization Study

**DOI:** 10.1210/clinem/dgaa250

**Published:** 2020-05-11

**Authors:** Jonathan Mark Fussey, Robin N Beaumont, Andrew R Wood, Bijay Vaidya, Joel Smith, Jessica Tyrrell

**Affiliations:** 1 Head and Neck Surgery, Royal Devon and Exeter Hospital, Exeter, UK; 2 Genetics of Complex Traits, College of Medicine and Health, University of Exeter, Exeter, UK; 3 Endocrinology, Royal Devon and Exeter Hospital, Exeter, UK

**Keywords:** mendelian randomization, thyroid neoplasms, obesity, diabetes mellitus, type 2, molecular epidemiology

## Abstract

**Background:**

The incidence of thyroid cancer is rising, and relatively little is known about modifiable risk factors for the condition. Observational studies have suggested a link between adiposity and thyroid cancer; however, these are subject to confounding and reverse causality. Here, we used data from the UK Biobank and Mendelian randomization approaches to investigate whether adiposity causes benign nodular thyroid disease and differentiated thyroid cancer.

**Methods:**

We analyzed data from 379 708 unrelated participants of European ancestry in the UK Biobank and identified 1812 participants with benign nodular thyroid disease and 425 with differentiated thyroid carcinoma. We tested observational associations with measures of adiposity and type 2 diabetes mellitus. One and 2-sample Mendelian randomization approaches were used to investigate causal relationships.

**Results:**

Observationally, there were positive associations between higher body mass index (odds ratio [OR], 1.15; 95% confidence interval [CI], 1.08-1.22), higher waist-hip ratio (OR, 1.16; 95% CI, 1.09-1.23), and benign nodular thyroid disease, but not thyroid cancer. Mendelian randomization did not support a causal link for obesity with benign nodular thyroid disease or thyroid cancer, although it did provide some evidence that individuals in the highest quartile for genetic liability of type 2 diabetes had higher odds of thyroid cancer than those in the lowest quartile (OR, 1.45; CI, 1.11-1.90).

**Conclusions:**

Contrary to the findings of observational studies, our results do not confirm a causal role for obesity in benign nodular thyroid disease or thyroid cancer. They do, however, suggest a link between type 2 diabetes and thyroid cancer.

Thyroid cancer is the most common endocrine malignancy, and its incidence is increasing, with rates expected to rise by 74% by 2035 in the United Kingdom (1). Along with childhood radiation exposure, a history of benign nodular thyroid disease is an important risk factor for differentiated thyroid cancer, suggesting that rather than representing distinct entities, benign nodules and thyroid cancers may fall on a spectrum of thyroid neoplasia (2, 3).

Among the frequently cited risk factors for malignancy in a patient presenting with a thyroid nodule is obesity (4), which is itself a growing public health concern. Large pooled analyses of case control studies (6796 and 848 932 participants, respectively) have demonstrated an association between body mass index (BMI) and thyroid cancer risk in men and women (5, 6), and 2 subsequent meta-analyses have corroborated these findings (7, 8). In addition, there is also some evidence from retrospective studies that obesity is associated with more aggressive features of thyroid cancers, including larger tumor size, extrathyroidal extension, more advanced tumor stage, and persistent disease following treatment (9-11). However, these studies tend to be observational, which carries an inherent risk of bias because of confounding factors and reverse causality. For example, obese patients may be more likely to undergo thyroid function screening than the general population, and tend to have higher TSH levels, which may be an independent risk factor for thyroid cancer (12). Efforts have been made to remove this bias by measuring BMI at the time of cytological analysis in previously undiagnosed patients with thyroid nodules, and found no association between BMI and thyroid cancer (13). Further complicating the link between obesity and thyroid cancer is the finding that type 2 diabetes mellitus (T2DM), a disease strongly associated with obesity, has been identified as a risk factor both for increased TSH levels (14) and thyroid cancer (15, 16).

The use of genetic epidemiology to unravel environmental determinants of disease relies on the fact that inheritance of genetic variants at conception is random and cannot be confounded by other risk factors. Mendelian randomization (MR; Fig. 1) uses genetic determinants of a trait such as obesity to test the hypothesis that the trait increases the risk of a disease (such as thyroid cancer) in the absence of bias from reverse causality and confounding (17).

**Figure 1.**
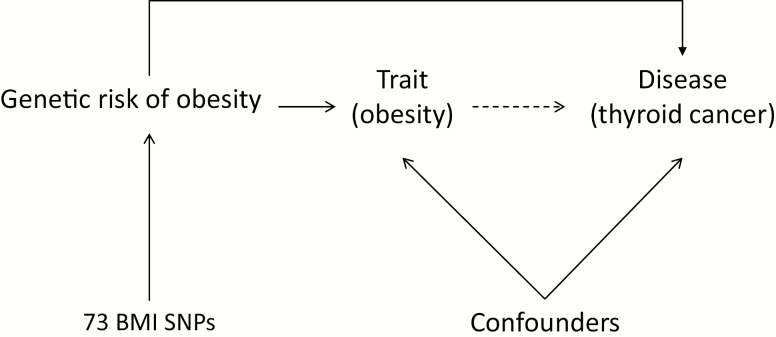
If a risk factor (e.g., body mass index) truly causes an outcome (e.g., thyroid cancer), then the genetic variants for the risk factor should also be associated with the outcome. Unlike the observed risk factor, the genetic variants are not susceptible to confounding by other risk factors as they are assigned at conception.

Genome-wide association studies (GWAS) have identified many polymorphisms associated with obesity (18), which can be used to construct individual genetic risk scores (GRS) and perform MR. Here, we test the hypothesis that obesity and other adiposity-related factors increase the risk of benign nodular thyroid disease and differentiated thyroid cancer. We used cancer registry data and clinical and genetic information from 379 708 unrelated participants of European ancestry in the UK Biobank to perform 1-sample MR, and 451 025 participants (without exclusion of related individuals) to perform 2-sample MR.

## Materials and Methods

### Participants

The UK Biobank is a longitudinal study of 500 000 participants between the ages of 40 and 69 recruited between 2006 and 2010 (19). Demographic and health-related information was obtained via questionnaires and interviews, and anthropometric measurements, blood pressure readings, and blood, urine, and saliva samples were taken at enrollment (20). Extensive health data are available via linkage with national cancer and death registries and the Hospital Episode Statistics database.

Genotyping was performed by Affymetrix (Santa Clara, CA) using DNA extracted from whole blood samples. Two specially designed single nucleotide polymorphism arrays with more than 95% content overlap were used: the UK Biobank Axiom array® was used for ~440 000 participants, and the UK BiLEVE Axiom array® for 50 000 participants (21). Sample quality control was performed by removing duplicated individuals, those identified as sex mismatches, of non-European descent, or outliers of heterozygosity, with an overall proportion of samples identified as poor quality of 0.2% (22).

### Exposure and outcome measures

To investigate the effect of obesity on the risk of developing benign nodular thyroid disease and thyroid cancer, a range of obesity-related exposure measures was used, including BMI, waist-hip ratio (WHR), and WHR adjusted for BMI. These were defined from the baseline measures, with BMI calculated from measured weight and height and WHR from measured waist and hip circumference. T2DM was also included as an exposure measure and was identified from the baseline questionnaire, as described previously (23). In brief, participants who had ever been told they had diabetes by a doctor were identified, then excluded if they were diagnosed under the age of 35, had received insulin within the first year of diagnosis, or were diagnosed less than a year before the date of enrollment to reduce the risk of inadvertently including participants with type 1 diabetes mellitus. Serum lipid levels and blood glucose were also used as biomarkers for metabolic health and their associations with benign thyroid disease and thyroid cancer were investigated.

Thyroid cancer cases were identified by the International Classification of Diseases-10 code C73 in the linked Cancer Registry data. Histology codes were used to refine the case definition. Specifically, participants were excluded if they had a diagnosis of medullary or anaplastic carcinoma, thyroid lymphoma, or unspecified histological subtypes because of the different etiology and pathophysiology of these subtypes. Finally, the date of first diagnosis was used to ensure that duplicated records and recurrences were excluded. This resulted in 638 cases including related individuals (and 425 after exclusion of related individuals). Benign nodular thyroid disease was defined using the International Classification of Diseases-10 code D34 from Hospital Episode Statistics data. Patients with toxic nodules were excluded. This resulted in 2149 cases, including related individuals (and 1812 after exclusion of related individuals). Date of diagnosis was used to exclude individuals diagnosed with both benign nodular thyroid disease and thyroid cancer within 12 months to exclude cases of diagnostic uncertainty. Separate control groups were used for the 2 cohorts to maximize comparability and reduce confounding, so that controls had no history of any cancer (n = 310 176) and no history of nodular thyroid disease (n = 377 896), respectively.

### Genetic data

Genetic variants for each potential risk factor were taken from published GWAS that excluded the UK Biobank. Seventy-three genetic variants associated with BMI were identified from the GIANT consortium study of up to 338 224 individuals (24), after exclusion of those not associated with BMI in all people of European ancestry, those reaching genome-wide levels of statistical significance in only 1 sex, and those known to be classified as a secondary signal within a locus. In a similar fashion, variants for WHR, WHR adjusted for BMI (adjWHR) (25) and T2DM risk (26) were identified. Finally, 14 common genetic variants associated a high body fat percentage but low metabolic disease risk, as identified by Yaghotkar et al (27) were used as favorable adiposity variants. Weighted GRS for each potential risk factor trait were produced using the size of the effect of each variant as reported in the primary GWAS and rescaled according to the number of trait-causing alleles. All genetic risk scores strongly associated with their corresponding traits, with all F statistics >10.

### Statistical analysis of observational data

Initial analyses using logistic regression models were performed to ascertain demographic differences between cases and controls. We then investigated associations between clinical measures of obesity and related metabolic parameters (including serum high-density lipoprotein [HDL], low-density lipoprotein [LDL], triglycerides, and glucose), and thyroid cancer and benign nodular thyroid disease using logistic regression models. These models included age, sex, smoking status, alcohol consumption, Townsend deprivation index, BMI, and T2DM as covariates. Only factors found to be associated with either benign nodular thyroid disease or thyroid cancer in these observational analyses were taken forward for Mendelian randomization as outlined below.

### Statistical analysis of genetic data

MR (Fig. 1) uses genetic variants as instrumental variables, which are associated with an outcome only through their association with a particular risk factor (for example, measured BMI) (28). MR relies upon three assumptions: first, that the instrumental variable is associated with the risk factor of interest; second, that the instrumental variable is not affected by the confounding factors acting upon the association between the risk factor and outcome of interest; and, finally, that the instrumental variable is associated with the outcome of interest only via its effect on the modifiable risk factor.

One-sample MR was performed in 2 stages using the GRS. First, the strength of the instrumental variables was tested by regressing against the corresponding observed risk factors, using linear regression for continuous predictors and logistic regression for binary predictors, with adjustment for age, sex, assessment center, ancestral principal components, and genotyping platform. Predicted values and residuals from this regression model were saved (representing unconfounded estimates of variation in observed variants) and used in the second stage as the independent variable, with disease status as the dependent variable. Robust standard errors were used to allow for uncertainty in the estimate. Sensitivity analysis was performed by excluding patients with a background of hypothyroidism or hyperthyroidism (Table 1).

**Table 1.** Sensitivity Analysis for 1-sample MR with Exclusion of Patients with a Diagnosis of Hyperthyroidism or Hypothyroidism

Analysis	Exposure	OR Thyroid Cancer	95% CI	OR Benign Nodular Thyroid Disease	95% CI
Primary analysis	BMI	1.18	0.55-2.52	0.87	0.61-1.25
	WHR	1.45	0.55-3.85	1.52	0.93-2.50
	adjWHR	0.80	0.53-1.21	1.07	0.86-1.32
	T2DM	1.22	0.89-1.69	0.99	0.84-1.16
	FA	0.99	0.95-1.03	1.01	0.98-1.08
Sensitivity analysis	BMI	1.18	0.55-2.52	0.86	0.60-1.22
	WHR	1.45	0.55-3.85	1.55	0.94-2.54
	adjWHR	0.79	0.52-1.19	1.06	0.86-1.32
	T2DM	1.23	0.98-1.70	0.99	0.84-1.16
	FA	0.99	0.95-1.09	1.01	0.99-1.04

adjWHR, WHR adjusted for BMI; BMI, body mass index; CI, confidence interval; FA, favourable adiposity; OR, odds ratio; T2DM, type 2 diabetes mellitus; WHR, waist-hip ratio.

Two-sample MR was also performed in a larger related subsample (n = 451 025). Here, GWAS of the thyroid cancer and benign nodular thyroid disease variables were performed using BOLT-LMM software to correct for inter-relatedness (29). Known genetic variants for our predictor traits of interest were then extracted and 3 different methods of 2-sample MR performed. First, inverse variant weighted instrumental variable analysis. Inverse variant weighted assumes that all genetic instruments are valid and is therefore susceptible to horizontal pleiotropy whereby variants have an effect on the outcome via a route other than the risk factor of interest. To reduce this potential source of bias, we also used the MR-Egger and Median MR techniques (30, 31), which are more robust to pleiotropy. In MR-Egger analysis, the intercept is unconstrained to remove the assumption that all variants are valid instrumental variables and allow a weighted regression. This reduces the possibility of variants having a stronger effect on the outcome than the exposure trait. Median-MR uses the median instrumental variable from all included variants and allows for up to 50% of the variants to be invalid, and thus is also more resistant to pleiotropy.

## Results

Basic demographic information on included participants and controls is displayed in Table 2. Participants in both the benign nodular thyroid disease and thyroid cancer groups were more likely to be female and older at recruitment than controls. Other demographics including smoking status, Townsend Deprivation Index, alcohol intake, mean BMI, and WHR were not different between the thyroid cancer and control groups. There were, however, differences in the benign nodular thyroid disease group, with cases more likely to be obese, more likely to have T2DM, less likely to be current smokers, have a lower alcohol intake, be more deprived (based on the Townsend Deprivation Index), and have a lower WHR. Of the 425 patients with a diagnosis of differentiated thyroid cancer, 117 had a previous history of benign nodular thyroid disease.

**Table 2.** Summary of Demographic Characteristics of Included Participants

Characteristic	Benign Nodular Thyroid Disease	Controls	*P* Value	Thyroid Cancer	Cancer-free Controls	*P* Value
N	1812	377 896		425	310 176	
Female	1492 (82.34)	203 244 (53.78)	<1 × 10^-15^	316 (74.35)	165 537 (53.37)	5.6 × 10^-17^
Mean age at recruitment (SD), years	58.92 (7.39)	57.23 (8.01)	<1 × 10^-15^	57.72 (7.29)	56.54 (8.04)	0.0022
Smoking status						
Never	976 (53.86)	203 242 (53.78)	0.04	233 (54.82)	169 785 (54.74)	0.40
Former	649 (35.82)	133 744 (35.40)		152 (35.76)	107 027 (34.51)	
Current	166 (9.16)	35 774 (9.47)		32 (7.53)	29 261 (9.43)	
Missing	21 (1.16)	5136 (1.36)		8 (1.88)	4103 (1.32)	
Mean Townsend Deprivation index (SD)	-1.16 (3.09)	-1.48 (2.99)	4.8 × 10^-8^	-1.63 (2.94)	-1.45 (2.99)	0.42
Inverse normalized mean units of alcohol per week (SD)	0.20 (1.07)	0.57 (1.04)	2.5 × 10^-6^	0.37 (1.00)	0.57 (1.04)	0.48
Mean BMI (SD), kg/m^2^	27.96 (5.23)	27.38 (4.78)	4.1 × 10^-12^	27.55 (5.34)	27.38 (4.77)	0.16
Obese: BMI 30-40 kg/m^2^	471 (25.99)	83 503 (22.10)	1.6 × 10^-9^	111 (26.12)	68 563 (22.10)	0.068
Mean waist hip ratio (SD)	0.85 (0.087)	0.87 (0.090)	1.4 × 10^-9^	0.85 (0.90)	0.87 (0.09)	0.083
Type 2 diabetes	70 (3.86)	11 996 (3.17)	0.0037	16 (3.76)	9290 (3.0)	0.16

Values stated are numbers (percentages), unless otherwise stated. *P* values calculated using logistic regression adjusted for age and sex.

BMI, body mass index; SD, standard deviation.

### Observational associations

#### Benign nodular thyroid disease.

 Higher adiposity was associated with a higher odds of benign nodular thyroid disease, for example obese patients (defined by a BMI between 30 and 40 kg/m^2^) had a 1.38 higher odds (95% confidence interval [CI], 1.17-1.62) than those with a normal BMI, and each 1 standard deviation increase in BMI (4.8 kg/m^2^) resulted in a 1.15 higher odds of benign nodular thyroid disease (95% CI, 1.08-1.22). WHR and adjWHR were associated with benign nodular thyroid disease, although some sex differences were observed, with stronger associations in men than in women (Tables 3 and 4). T2DM was not associated with benign nodular thyroid disease (Table 3).

**Table 3.** Observational Associations Between Measured Traits and Benign Nodular Thyroid Disease and Thyroid Cancer

Trait	OR Benign Nodular Disease	95% CI	*P* Value	OR Thyroid Cancer	95% CI	*P* Value
Obesity (BMI 30-40 kg/m^2^)	1.38	1.17-1.62	1.2 × 10^-4^	1.34	0.99-1.84	5.7 × 10^-2^
BMI	1.15	1.08-1.22	8.0 × 10^-6^	1.02	0.91-1.15	0.72
WHR	1.16	1.09-1.23	1.8 × 10^-6^	1.12	1.00-1.26	5.6 × 10^-2^
adjWHR	1.11	1.04-1.18	8.8 × 10^-4^	1.13	1.00-1.27	4.8 × 10^-2^
T2DM	1.04	0.71-1.5	0.85	1.03	0.48-2.20	0.95
Serum HDL	0.76	0.63-0.93	7.4 × 10^-3^	0.68	0.45-1.00	5.3 × 10^-2^
Serum LDL	0.89	0.83-0.96	1.4 × 10^-3^	1.00	0.87-1.15	0.97
Serum triglycerides	0.96	0.90-1.03	0.3	1.06	0.93-1.20	0.39
Serum glucose	0.97	0.90-1.05	0.46	0.97	0.83-1.14	0.75

Continuous variables are reported as odds ratio per 1 standard deviation higher predictor. *P* values calculated using logistic regression with age, sex, smoking status, alcohol consumption, Townsend deprivation index, BMI, and T2DM as covariates.

adjWHR, WHR adjusted for BMI; BMI, body mass index; CI, confidence interval; HDL, high-density lipoprotein; LDL, low-density lipoprotein; OR, odds ratio; T2DM, type 2 diabetes mellitus. WHR, waist-hip ratio.

**Table 4.** Observational and 1-sample MR Associations Between BMI, WHR, and Type 2 Diabetes and Benign Nodular Thyroid Disease and Thyroid Cancer in Men and Women

Characteristic	OR Benign Nodular Disease	95% CI	*P* Value	OR Thyroid Cancer	95% CI	*P* Value
Female BMI						
Measured	1.18	1.10-1.26	4.2 × 10^-6^	1.02	0.89-1.18	0.74
1-sample MR	0.93	0.63-1.38	0.71	0.87	.036-2.09	0.75
Male BMI						
Measured	1.06	0.92-1.21	0.42	1.02	0.82-1.27	0.87
1-sample MR	0.80	0.34-1.88	0.6	3.13	0.73-13.43	0.12
Female WHR						
Measured	1.14	1.07-1.22	1.2x10^4^	1.05	0.92-1.22	0.46
1-sample MR	1.45	0.85-2.50	0.17	1.05	0.33-3.30	0.94
Male WHR						
Measured	1.22	1.07-1.40	3.1 × 10^-3^	1.30	1.05-1.62	1.7 × 10^-2^
1-sample MR	1.69	0.50-5.66	0.4	3.67	0.56-24.12	0.18
Female WHR adjusted for BMI						
Measured	1.08	1.01-1.16	2.1 × 10^-2^	1.04	0.91-1.20	0.57
1-sample MR	1.00	0.79-1.26	0.97	0.74	0.45-1.20	0.22
Male WHR adjusted for BMI						
Measured	1.20	1.06-1.37	5.6 × 10^-3^	1.34	1.09-1.67	6.5 × 10^-3^
1-sample MR	1.36	0.80-2.29	0.25	1.04	0.46-2.33	0.93

*P* values calculated using logistic regression adjusted for age, with smoking status, alcohol consumption, Townsend deprivation index, BMI, and T2DM used as covariates for measured values.

adjWHR, WHR adjusted for BMI; BMI, body mass index; CI, confidence interval; HDL, high-density lipoprotein; LDL, low-density lipoprotein; OR, odds ratio; T2DM, type 2 diabetes mellitus. WHR, waist-hip ratio.

Measured serum HDL and LDL levels were both associated with a lower odds of benign nodular thyroid disease; however, there was no association with serum triglycerides or glucose levels (Table 3).

#### Thyroid cancer.

Obesity was trending toward higher odds of thyroid cancer (odds ratio [OR], 1.34; 95% CI, 0.99-1.84), as was higher WHR (OR, 1.12; 95% CI, 1.00-1.26) and adjWHR (OR, 1.13; 95% CI, 1.00-1.27; Table 3), but in all individuals a per unit higher BMI was not associated with thyroid cancer. There was no association between T2DM and thyroid cancer.

As in benign nodular thyroid disease, a higher serum HDL level was trending toward a lower odds of thyroid cancer, with each 1 standard deviation (0.38 mmol/L) higher HDL associating with 0.68 lower odds of thyroid cancer (95% CI, 0.45-1.00). LDL, triglyceride levels, and serum glucose were not associated with thyroid cancer (Table 3).

### Mendelian randomization

#### Benign nodular thyroid disease.

**Figure 2.**
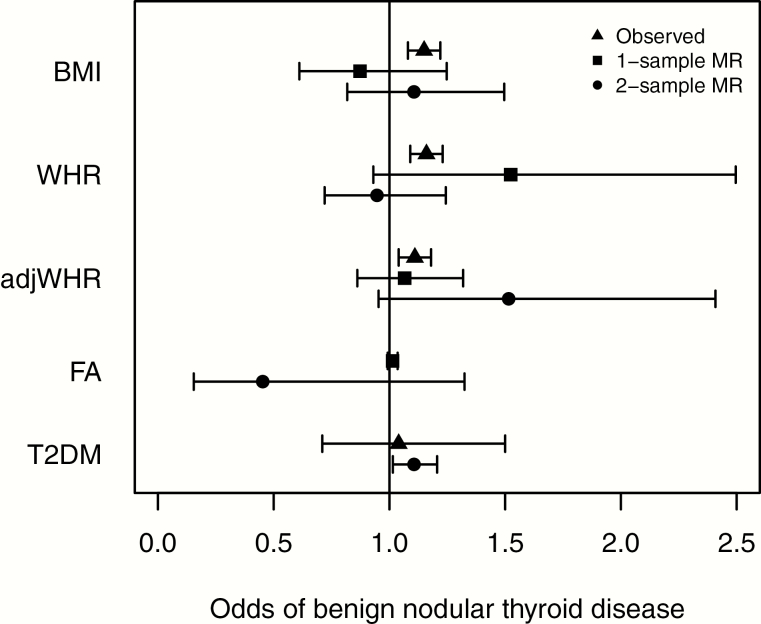
Forest plot showing observed, 1 sample MR and 2 sample MR (instrumental variable analysis) associations between BMI, WHR, adjWHR, favorable adiposity, T2DM, and benign nodular thyroid disease. Odds ratios and 95% confidence intervals are shown (there are no observational estimates for favorable adiposity because this phenotype of higher adiposity and lower risk of metabolic disease can only be tested in this context with genetic variants. One-sample MR was not performed for T2DM because of the difficulty interpreting results using binary variables). adjWHR, WHR adjusted for BMI; BMI, body mass index; MR, Mendelian randomization; T2DM, type 2 diabetes mellitus; WHR, waist-hip ratio.

There was some evidence that a higher genetic liability to T2DM caused benign thyroid disease (OR, 1.11; 95% CI, 1.01-1.21; Fig. 2 and Table 5). The effect estimate was consistent across the more pleiotropy-resistant methods, but the CIs crossed the null. One-sample MR was not performed for T2DM because of the difficulty in interpreting MR using binary variables. There was no evidence that BMI, WHR, or favorable adiposity cause benign nodular thyroid disease; however, in all cases the CIs overlapped the observational estimates (Fig. 2). There was tentative evidence for a causal relationship between genetically instrumented higher WHR adjusted for BMI and benign nodular thyroid disease (OR, 1.51; 95% CI, 0.95-2.41; *P* = 0.085). One-sample MR did not reveal evidence of a causative role for HDL or LDL in benign nodular thyroid disease; however, it revealed a protective effect of serum triglycerides (TG; OR, 0.69; 95% CI; 0.53-0.91). Two-sample MR did not demonstrate any associations between biomarkers and benign thyroid disease.

**Table 5.** Results of 2-sample MR Analysis for Benign Nodular Thyroid Disease

	IVW		Egger			WM		PWM	
Trait	OR (95% CI)	*P* Value	OR (95% CI)	*P* Value	Int p	OR (95% CI)	*P* Value	OR (95% CI)	*P* Value
BMI	1.10 (0.82-1.50)	0.51	0.59 (0.31-1.12)	0.11	0.03	0.67 (0.43-1.01)	0.07	0.67 (0.43-1.04)	0.08
WHR	0.95 (0.72-1.24)	0.69	1.06 (0.48-2.33)	0.89	0.76	0.90 (0.61-1.33)	0.59	0.90 (0.60-1.34)	0.60
WHR adjusted for BMI	1.52 (0.95-2.41)	0.08	0.54 (0.11-2.60)	0.45	0.18	1.06 (0.62-1.83)	0.83	1.06 (0.63-1.80)	0.82
Favorable adiposity	0.45 (0.15-1.32)	0.17	0.02 (0.00-0.32)	0.02	0.04	0.43 (0.13-1.46)	0.18	0.42 (0.12-1.52)	0.19
T2DM	1.11 (1.01-2.21)	0.02	1.12 (0.95-1.33)	0.17	0.84	1.11 (0.97-1.26)	0.12	1.11 (0.98-1.27)	0.11

Odds ratios, 95% CIs, and *P* values for the inverse variant weighted (IVW), Egger, weighted median (WM), and penalized weighted median (PWM) analyses are displayed. Int *P* represents the p intercept of Egger analysis, which is a measure of horizontal pleiotropy.

BMI, body mass index; CI, confidence interval; WHR, waist-hip ratio.

#### Thyroid cancer.

**Figure 3.**
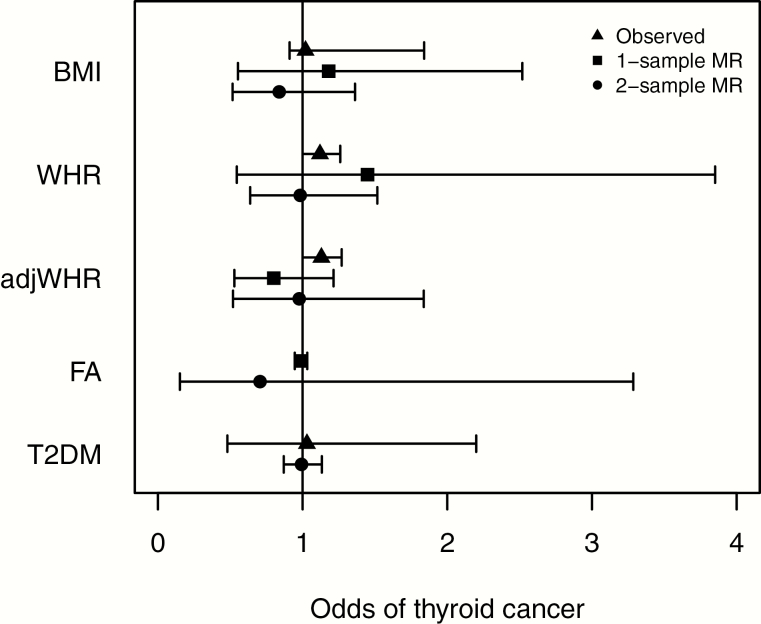
Forest plot showing observed, 1 sample MR and 2 sample MR (instrumental variable analysis) associations between BMI, WHR, adjWHR, favorable adiposity, T2DM, and thyroid cancer. Odds ratios and 95% confidence intervals are shown (there are no observational estimates for favorable adiposity because this phenotype of higher adiposity and lower risk of metabolic disease can only be tested in this context with genetic variants. One-sample MR was not performed for T2DM because of the difficulty interpreting results using binary variables). adjWHR, WHR adjusted for BMI; BMI, body mass index; MR, Mendelian randomization; T2DM, type 2 diabetes mellitus; WHR, waist-hip ratio.

MR analysis provided no evidence for a causal role for BMI, WHR, adjWHR, or favorable adiposity, according to neither raw GRS (Fig. 3; Table 6) nor when analyzed by quartile for GRS. Although conventional MR approaches did not provide evidence that a higher genetic liability for T2DM caused thyroid cancer, individuals in the top 25% of the GRS for T2DM were at higher odds of thyroid cancer (OR, 1.45; 95% CI, 1.11-1.90; Table 7). Using 1- and 2-sample MR, no causative role was found for LDL, HDL, or TG levels.

**Table 6.** Results of 2-sample MR Analysis for Thyroid Cancer

	IVW		Egger			WM		PWM	
Trait	OR (95% CI)	*P* Value	OR (95% CI)	*P* Value	Int P	OR (95% CI)	*P* Value	OR (95% CI)	*P* Value
BMI	0.84 (0.52-1.36)	0.48	0.70 (0.24-2.01)	0.51	0.71	1.04 (0.46-2.34)	0.93	1.04 (0.46-2.32)	0.93
WHR	0.98 (0.64-1.52)	0.94	1.46 (0.42-5.11)	0.55	0.51	1.04 (0.53-2.04)	0.91	1.07 (0.56-2.04)	0.84
WHR adjusted for BMI	0.98 (0.52-1.84)	0.94	0.51 (0.06-4.34)	0.54	0.54	1.27 (0.51-3.17)	0.61	1.32 (0.54-3.20)	0.54
Favorable adiposity	0.71 (0.15-3.29)	0.66	1.89 (0.02-162.9)	0.78	0.63	0.76 (0.09-6.53)	0.80	0.76 (0.09-6.65)	0.80
T2DM	0.99 (0.87-1.13)	0.91	0.92 (0.71-1.81)	0.50	0.43	0.94 (0.76-1.17)	0.58	0.94 (0.76-1.71)	0.59

Odds ratios, 95% CIs, and *P* values for the inverse variant weighted (IVW), Egger, weighted median (WM) and penalized weighted median (PWM) analyses are displayed. Int *P* represents the p-intercept of Egger analysis, which is a measure of horizontal pleiotropy.

BMI, body mass index; CI, confidence interval; WHR, waist-hip ratio.

**Table 7.** Odds ratios for Thyroid Cancer by Quartiles of Genetic Risk of T2DM

	No. (%)			
Quartiles	Cases	Controls	OR^*a*^ (95% CI)	*P*
1	89 (20.9)	77 358 (24.9)	1.00	
2	101 (23.8)	77 529 (24.9)	1.14 (0.85-1.51)	0.38
3	106 (24.9)	77 701 (25.1)	1.19 (0.90-1.57)	0.23
4	129 (30.4)	77 588 (25.0)	1.45 (1.11-1.90)	0.0068

^*a*^Odds ratios and *P* values adjusted for age, sex ancestral principal component, and assessment center with a logistic regression model.

## Discussion

### Principal findings

This study examined the causal role of adiposity and related traits in both benign nodular thyroid disease and thyroid cancer, and started by examining observational associations between measured traits and both outcomes. There was no evidence of an association between observed BMI and thyroid cancer among UK Biobank participants, although there was a positive association between observed BMI and both WHR and WHR adjusted for BMI and benign nodular thyroid disease.

Other published observational studies have reported somewhat inconsistent results regarding obesity and thyroid cancer. Positive associations have been reported by pooled analysis of retrospective cohort studies (4) and meta-analyses (7, 8). However, these analyses relied on a heterogeneous group of studies with varying levels of adjustment for confounding factors such as a previous diagnosis of benign nodular thyroid disease, which is in itself a known risk factor for the development of thyroid cancer (3). Furthermore, a prospective study focusing on a group of patients known to have thyroid nodules measured their BMI at the time of cytological analysis and found an inverse relationship between obesity and malignant features on cytology, and no relationship when considering a smaller subset of patients with surgical histology available (13).

Using MR, we did not find evidence of a causal link between obesity and benign nodular thyroid disease to support our observational findings. This could be due to confounding factors such as T2DM or TSH levels that increase both the risk of benign nodular thyroid disease and obesity, resulting in false associations when using clinical observations, which are limited by using genetic variants. In addition to this, the MR findings do not support a causal role for obesity in thyroid cancer.

MR provided some evidence for a causal link between T2DM and benign nodular thyroid disease, which supports the observational association in the UK Biobank. Although there was no observational association between T2DM and thyroid cancer, the patients in the top quartile for genetic risk of T2DM did have a significantly higher odds of thyroid cancer, suggesting a possible causative role.

Previous observational studies investigating the link between T2DM and thyroid cancer have identified a positive association (15, 16). This along with the negative findings for obesity-related risk factors suggests that type 2 diabetes may be a risk factor for thyroid cancer independent of obesity. The mechanisms for this association are not clear; however, IGF-1 receptors are overexpressed on thyroid cancer cells and may be activated by chronically elevated levels of circulating insulin, leading to cell proliferation (16). This theory is supported by the finding that thyroid nodules are associated with insulin resistance (32). Further work is required to confirm the causal role of T2DM; however, if confirmed, it could have implications for risk stratification and management of indeterminate nodules in diabetic patients.

We also investigated the association between serum lipid levels and both benign nodular thyroid disease and thyroid cancer, using observed levels as well as genetically instrumented levels. We found measured HDL and LDL to be inversely associated with odds of benign nodular thyroid disease; however, the findings of the MR analysis suggested no association between either HDL or LDL and benign nodular thyroid disease or thyroid cancer. MR did however suggest an unexpected protective role for higher TG levels in benign nodular thyroid disease but not thyroid cancer. This association needs further validation.

### Strengths and limitations

This represents the first MR study on the effect of adiposity and related traits on nodular thyroid disease and thyroid cancer; however, we acknowledge some limitations to our approach. First, we only had 425 thyroid cancer cases, which limits our power for both observational and MR analyses. The usefulness of observational analysis in particular is inevitably limited despite adjustment for known confounders in the statistical approach, which is why MR using genetic variables was used to further explore causal associations. Second, the UK Biobank is not population representative because participants were limited to adults between the ages of 40 and 69 living in the United Kingdom, with some evidence of healthy volunteer bias, resulting in lower rates of obesity and cancer incidence than age- and sex-matched members of the general population (33). Third, our definition of T2DM was based on self-reported T2DM, which is susceptible to recall bias. Although participants recently started on insulin, or diagnosed when younger than age 35 years were excluded to remove those with type 1 diabetes, it is inevitable that this approach will have resulted in the unnecessary exclusion of some participants with T2DM and perhaps the inclusion of a small number of participants with type 1 diabetes who were diagnosed at older than age 35 and who were not treated with insulin within a year of that diagnosis. Fourth, although we performed sensitivity analysis excluding patients with a diagnosis of hypothyroidism or hyperthyroidism, the diagnosis of benign nodular thyroid disease covers a wide range of conditions from single nontoxic nodule to multinodular goiter. Fifth, identification of diagnoses in biobanks relies upon accurate coding. Cancer registry coding is robust in the United Kingdom; however, coding of benign thyroid nodules is likely to be more sporadic, so our results are likely to underrepresent benign nodular thyroid disease within the UK Biobank population. Similarly, identification of controls relied on the lack of a record of the outcomes of interest in the cancer registry or HES registry rather than full clinical and radiological examination excluding them, which means that some participants with thyroid cancer or benign nodules will undoubtedly have been included in the control groups. It is reported that the prevalence of thyroid nodules on neck palpation is between 2% and 6%, and much higher on ultrasound (34). That the prevalence of benign thyroid nodules in the current study population was around 0.5% suggests that there was a significant number of participants with undiagnosed nodules. As in all biobank research, this results in a source of detection bias for control groups. The inadvertent inclusion of undiagnosed cases in the control group may weaken the associations identified both on observational analysis and MR analysis, thus disguising the true magnitude of the associations. Finally, the BMI GRS we used has been shown previously to be associated not only with BMI at enrollment in the UK Biobank, but also with self-reported obesity at the age of 10 (27). Other authors have suggested that body shape in middle age is a more important risk factor for thyroid cancer than body shape in early adulthood (35). The advantage of our BMI GRS is that it predicts high BMI over an individual’s lifetime, as opposed to the 1-time measurements used in many observational studies; however, the disadvantage is that it cannot discriminate causal effects of obesity at a particular time in life.

## Conclusions

The incidence of thyroid cancer is increasing and there is limited evidence for modifiable risk factors. Obesity and T2DM are both reported by observational studies as being risk factors, and although we were able to demonstrate evidence of a causative role for T2DM using MR, we were unable to find a causative link between obesity and either benign nodular thyroid disease or thyroid cancer. This suggests the possibility of other factors confounding the reported observational associations in the case of obesity. More work is needed to improve our understanding of the underlying mechanisms for the associations between thyroid nodules, thyroid cancer, and obesity.

## Abbreviations

adjWHR: adjusted waist-hip ratio
BMI: body mass index
CI: confidence interval
GRS: genetic risk score
GWAS: genome-wide association study
HDL: high-density lipoprotein
LDL: low-density lipoprotein
MR: Mendelian randomization
OR: odds ratio
T2DM: type 2 diabetes mellitus
TG: triglyceride
WHR: waist-hip ratio

## Additional Information

***Disclosure Summary:*** The authors have no conflicts of interest to declare.

***Data Availability:*** Restrictions apply to the availability of data generated or analyzed during this study to preserve patient confidentiality or because they were used under license. The corresponding author will on request detail the restrictions and any conditions under which access to some data may be provided.
